# Absence of Langerhans cells resulted in over-influx of neutrophils and increased bacterial burden in skin wounds

**DOI:** 10.1038/s41419-024-07143-1

**Published:** 2024-10-19

**Authors:** Zheng-Cai Wang, Yan-Yan Hu, Xiao Z. Shen, Wei-Qiang Tan

**Affiliations:** 1 Department of Plastic Surgery, Sir Run Run Shaw Hospital, Zhejiang University School of Medicine, Hangzhou, China; 2 Department of Physiology and Department of Cardiology of the Second Affiliated Hospital, Zhejiang University School of Medicine, Hangzhou, Zhejiang China

**Keywords:** Langerhans cells, Acute inflammation

## Abstract

Langerhans cells (LCs) are resident dendritic cells in the epidermis and their roles in presenting antigens derived from microorganisms present in the skin has been well appreciated. However, it is generally thought that incoming neutrophils are mainly responsible for eradicating invading pathogens in the early stage of wounds and a role of LCs in innate immunity is elusive. In the current study, we showed that wounds absent of LCs had a delayed closure. Mechanistically, LCs were the primary cells in warding off bacteria invasion at the early stage of wound healing. Without LCs, commensal bacteria quickly invaded and propagated in the wounded area. keratinocytes surrounding the wounds responded to the excessive bacteria by elevated production of CXCL5, resulting in an over-influx of neutrophils. The over-presence of activated neutrophils, possibly together with the aggravated invasion of bacteria, was detrimental to epidermal progenitor cell propagation and re-epithelialization. These observations underscore an indispensable role of LCs as effective guardians that preclude both bacteria invasion and damages inflicted by secondary inflammation.

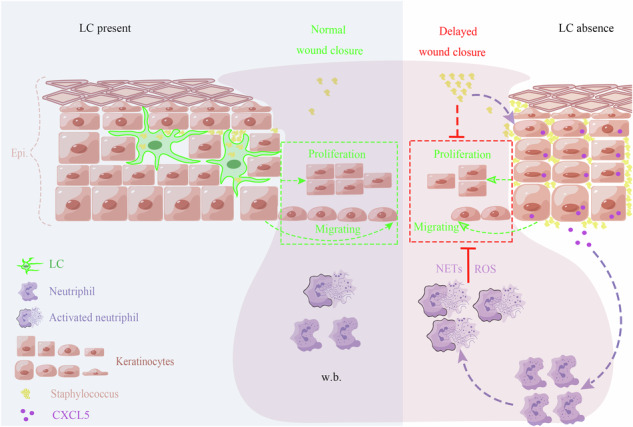

## Highlights

- Langerhans cells (LCs) are considered resident dendritic cells in the epidermis and their roles in presenting antigens derived from microorganisms present in the skin has been well appreciated.
- However, the role of LCs in wound healing is controversial.
- In this study, we demonstrate that wounds absent of LCs had a delayed closure.
- Mechanistically, LCs may be the primary cells in warding off bacteria invasion at the early stage of wound healing.
- Without LCs, propagation of commensal bacteria was evident in the early stage of wounds, which stimulated keratinocytes to overproduce CXCL5, a neutrophil chemokine. Over-influx of 3neutrophils significantly slowed epidermal progenitor cell propagation, re-epithelization and wound healing in the absence of LCs.

## Introduction

Epithelium is a tissue featured for its direct exposure to the external environment and the associated various commensal and pathogenic microorganisms and noxious agents [1]. In terms of skin, it is the largest organ of human body and forms a protective shield against microbial invasion and a variety of environmental inflictions [1]. As such, epidermis, the skin epithelium, has a relatively fast turnover rate owing to continuous wear and tear. Stem and progenitor cells residing in the bottom layer of skin epithelium actively replace the terminally differentiated but wearing keratinocytes on top of them [2]. During wounding, skin re-epithelization also involves collective migration of keratinocytes across the injured dermis [3].

Formation of a proper epithelial barrier after acute injury requires a coordinated action and cross-talk of keratinocytes and immune cells at the wound site. For example, impacts of neutrophils in wound healing can be viewed as a double-edged sword [4]. Too few neutrophils risks infection and delayed epithelium recovery [5], whereas over-persistence of neutrophils in injured tissues also delays epithelium healing through collateral tissue damage by inflammatory effectors [6]. Therefore, neutrophil recruitment and activation in wounding site is tightly tailored, a regulation process has not been clearly addressed. Langerhans cells (LCs) inhabit the epidermis and account for a majority of immune cells in the skin epidermis in resting state. The localization of LCs suggests that these immune cells will be one of the first immune cell types to interact with substances and infectious agents invading the skin, especially in case of wounding. On the other hand, keratinocytes which are the epithelial cells in the skin must also sense and respond to environmental assaults as well. Substantial evidence has emerged and suggested that epithelial cells would respond to the encounter of microorganisms by secreting various cytokines and chemokines so as to orchestrate orderly immune responses [7]. However, how keratinocytes and LCs, the two resident epidermal cells, cope with invading microorganisms under skin injury is not fully understood.

In the current study, we revealed a capacity of LCs in preventing the spreading of skin symbiont bacteria into the wounds by a direct phagocytosis-facilitated removal on encounter. Without LCs, propagation of commensal bacteria was evident in the early stage of wounds, which stimulated keratinocytes to overproduce CXCL5, a neutrophil chemokine. Over-influx of neutrophils significantly slowed epidermal progenitor cell propagation, re-epithelization and wound healing in the absence of LCs. Here, we delineate an innate immune “gatekeeper” function of LCs against microorganisms in addition to their well-appreciated role in presenting alien antigens. This action was crucial for a timely wound healing since it at the same time precluded unnecessary over-inflammation which by itself was toxic to wound healing.

## Results

### Deficiency of Langerhans cells slows re-epithelialization of wounds following injury

To investigate a role of LCs in wound healing, we employed a mouse line expressing diphtheria toxin receptor downstream of langerin (CD207) promoter (hereafter *Langerin*^DTR^). In the skin, langerin is expressed in almost all of the epidermal LCs [8] and the majority of classical type 1 dendritic cells (cDC1) which reside in dermis [9], as further verified by our flow cytometry analysis of the langerin^+^ reporter *Langerin*^EGFP^ mice (Fig. S1). Of note, it has been demonstrated in both human and mouse that the density of LCs is about 10–20 folds higher than the underlying dermal langerin^+^ DCs [10]. Treating *Langerin*^DTR^ mice with two shots of 1 μg DT (2 μg in total) in 4 days, a regimen used in a previous study to investigate wound healing [11], could almost completely deplete both epidermal LCs and dermal langerin^+^ DCs (Fig. S2A). Moreover, this dose induced a marked increase of pro-inflammatory cytokines in the skin (Fig. S2B), accompanied by a profound TUNEL^+^ apoptotic profile of tissue in the dermis (Fig. S2C), which would confound mechanism interpretation of wound healing studies. In contrast, when we applied a much lower dose (100 ng) of DT to the *Langerin*^DTR^ mice, one shot induced the removal of about 57.8% of langerin^+^ cDC1 but could achieve a near-complete depletion of LCs (Figs. S2A). More importantly, this low dose did not induce apparent inflammation and tissue cell apoptosis (Figs. S2B and S2C). In a temporal study, one shot of the low dose of DT could deplete LCs in 24 h (Fig. S2D, S2E) and this effect could last for at least 5 d (Fig. S2D). Therefore, we stick to a protocol of the low dose (100 ng) of DT once every 5 d in the following study.

**Fig. 1:**
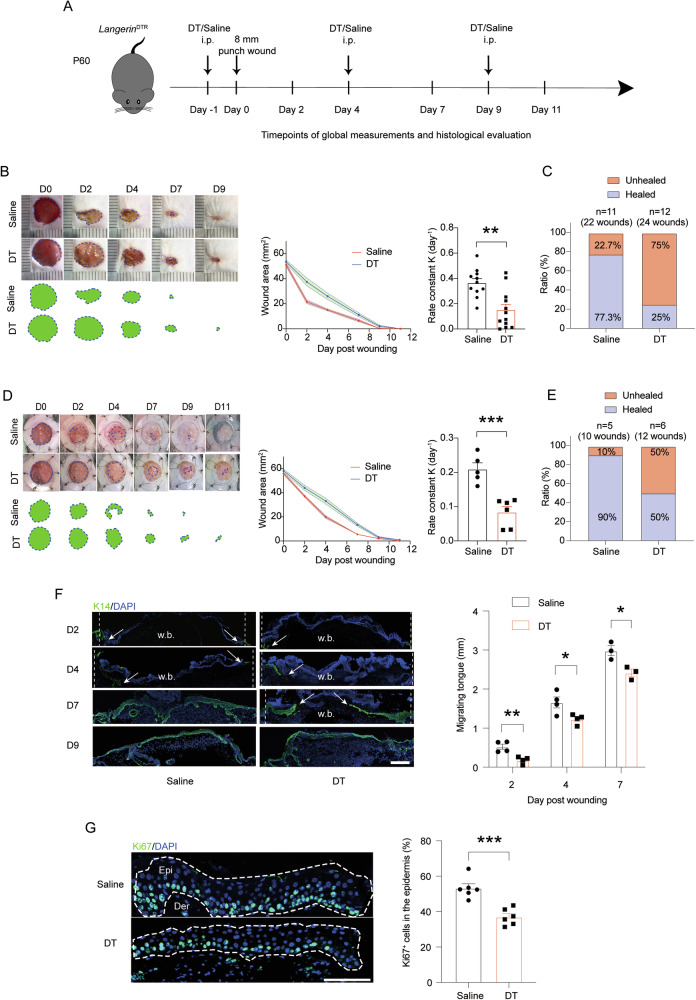
Delayed wound repair after depletion of langerin^+^ cells. **A**. *Langerin*^DTR^ mice were i.p. treated with saline (as controls) or 100 ng DT every 5 d. One day after the first treatment, dorsal skin full-thickness wounds were made followed by observation in the indicated time points. **B** (left) Representative pictures of wounds. The green area circled by dotted line represents the unhealed wound area. (middle) Kinetic statistics of wound area change over time. (right) Curve fitting rate calculated based on the unhealed wound area over the observation period. *n* = 11 for saline group and n = 12 for DT group. **C** Frequencies of healed and unhealed wounds on the 9th day after wounding. **D**, **E** The above full-skin wounding model was performed on *Langerin*^DTR^ mice affixed with a silicone splint. **D** Representative pictures of wounds (left), kinetic statistics of wound area changes over time (middle), and curve fitting rate (right). *n* = 5 for saline group and *n* = 6 for DT group. **E** Frequencies of healed and unhealed wounds on the 11th day after wounding. **F** Migration of epidermal progenitor cells (K14^+^) of the wound edge during the observation period. The dotted lines indicate the initial wound edge, and arrows mark wound tongues. w.b., wound bed. Scale bar: 100 μm. **G** The percentage of Ki67^+^ cells among all epidermal cells in the proliferating hub area (1 mm away from the wound edge). the white dotted area indicated the epidermis. Scale bar: 100 μm. *P < 0.05, **P < 0.01, ***P < 0.001 by two-tailed unpaired *t* test. Data are depicted as mean ± SEM. Data are derived from at least 2 independent experiments.

We next challenged the skin to wounding, to see if the deficiency of langerin^+^ cells, especially the LCs, would affect an injury response and wound healing. *Langerin*^DTR^ mice were treated with DT or saline (as control), and a dorsal skin full-thickness wounding model, i.e., 8 mm epidermis+dermis wounds, was created by punch biopsies on the animals’ backs. After that, we measured the wound size at intervals over 11 d by which a full closure could be reached in most mice (Fig. 1A). As shown in the images from a representative experiment, the DT-treated mice closed their wounds ~2.4 times slower than their saline-treated littermates (Fig. 1B). When quantified over 3 independent wound studies, the most noticeable difference was evident during day 2~4 post wounding. Consistent with a delay of healing, while ~77.3% of control wounds completed closure by 9 d, only ~25% of LC-deficient wounds reached this goal in the same time frame (Fig. 1C). Of note, DT by itself did not alter the wound healing process in C57BL/6 mice (Fig. S3).

To exclude myofibroblast-mediated dermal contraction as a notable contributor to the wound closure, we next affixed a silicone splint around full-thickness wounds. While the wound closure rates declined in both groups relative to their non-splinted counterpart groups, the difference between LC-depleted and LC-intact skin was maintained, as the wound repair process in the LC-depleted mice was over twice as slow as in the controls (Fig. 1D). As such, while ~90% of control wounds completed closure by 11 days, only ~50% of LC-deficient wounds reached this goal in the same time frame (Fig. 1E). These data suggest that the wound healing delay could be attributed in a great part to a slowed re-epithelization process. After a full-thickness wound, re-epithelialization is mediated by sensitized epidermal progenitor cells which are housed in the innermost (basal) layer of skin epithelium and are distinguishable by expressing basal keratin 14 (K14) [12]. Indeed, LC-deficient skin displayed delayed re-coverage of epidermal progenitor cells on wounded area, manifested by a slower extension of K14^+^ epithelial tongue (Fig. 1F). The migrating keratinocytes were within a distance of 500 μm from the wound front, and the epidermal proliferating hubs locate between 500 and 1500 μm from the wound front [3]. Consistent with a slower extension of healing edge, there were fewer Ki67^+^ keratinocytes at the basal layer of epidermis in the proliferating hub area of the LC-deficient mice on day 2 (Fig. 1G). These results suggest that a normal distribution of skin langerin^+^ cells is required for epithelial progenitor cells to react promptly to wound formation.

**Fig. 2:**
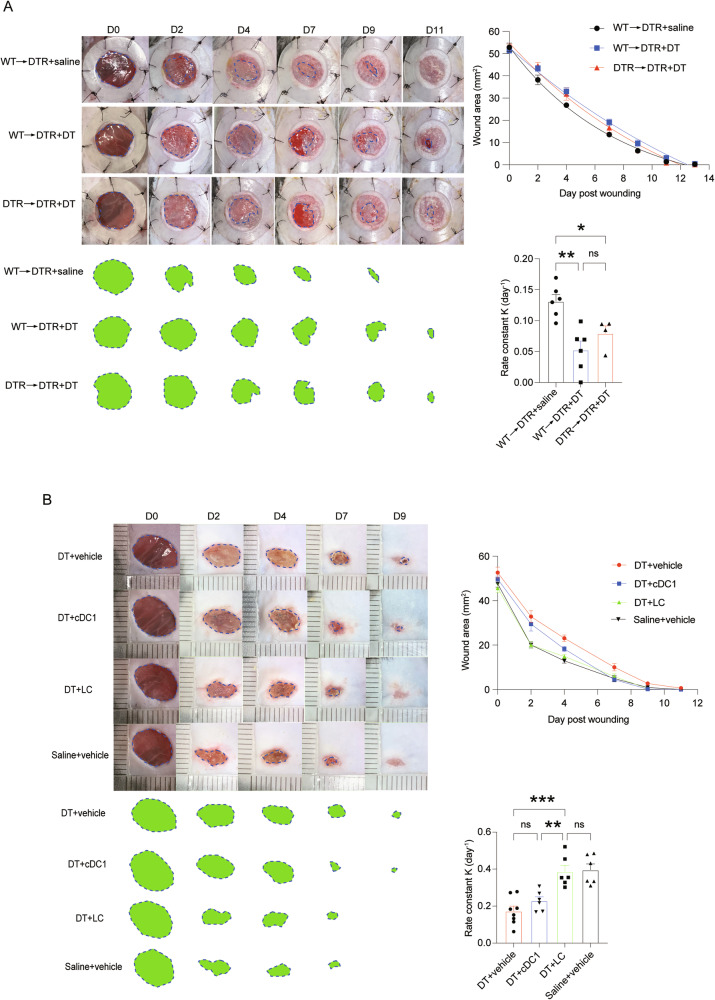
LC deficiency is the cause of delayed wound healing after DT injection into *Langerin*^DTR^ mice. **A** Representative pictures of wounds, kinetic statistics of wound area changes over time, and curve fitting rate of the *Langerin*^DTR^ mice transplanted with the indicated BM in a silicone affixed wound model. The green area circled by dotted line represents the unhealed wound area. **B** *Langerin*^DTR^ mice treated with DT or saline, and a full-skin wound model was performed 1 day later (silicone splint was not used since it would affect the transferred cells). Right after wounding, 5 × 10^4^ LCs or 5 × 10^3^ cDC1 cells were adoptively transferred into the wound periphery (the number of transferred cells were estimated according to the normal number of LCs and cDC1 distributed in the wounding area). Representative pictures of wounds, kinetic statistics of wound area changes over time, and curve fitting rate were shown. ns not significant. *P < 0.05, **P < 0.01, ***P < 0.001 by ordinary one-way ANOVA test. Data are depicted as mean ± SEM. Data are derived from at least 2 independent experiments.

Since a portion of cDC1s were also affected by DT treatment in the *Langerin*^DTR^ mice, to distinguish the roles of LCs and cDC1s in wound healing, we next exploited the difference of them in radioresistance. LCs are radioresistant and long-lived, whereas classical DCs are short-lived and rely on continuous replenishment from bone marrow (BM) which are radiosensitive [13]. After lethal irradiation, the *Langerin*^DTR^ mice were reconstituted with the BM from wild-type (WT) or *Langerin*^DTR^ donors. Eight weeks after BM transplantation when peripheral myeloid cells restored normalcy, the recipients were treated with DT. The WT BM-reconstituted *Langerin*^DTR^ mice exhibited near-complete loss of LCs but with intact dermal cDC1 distribution (Fig. S4), confirming specific depletion of LCs by this strategy. Compared to the WT→*Langerin*^DTR^ mice treated with saline, both WT→*Langerin*^DTR^ and *Langerin*^DTR^→*Langerin*^DTR^ groups showed delayed wound healing after being treated with DT and there was no statistical difference between the DT-treated groups in their healing rates (Fig. 2A). In a parallel experiment, we did another cohort of BM transplantation by reconstituting WT mice with *Langerin*^DTR^ BM. After BM recovery, these recipients were treated with DT or saline and then were subject to wounding. With this strategy, DT treatment would specifically affect langerin^+^ cDC1 but spare LCs. It displayed that DT treatment had little influence on wound healing rate (Fig. S5A). Thus, the BM transplantation experiments underscore an indispensable role of LCs in wound healing. Based on the difference in replenishment speed between LC and cDC1, we injected *Langerin*^DTR^ mice with saline or DT. 30 days later, cDC1 had fully repopulated the dermis, whereas LCs remained absent [13]. In this scenario, DT injection resulted in delayed wound healing (Fig. S5B), similar to our previous results (Fig. 1B, D), which further highlights the essential role of LCs in wound healing, as opposed to cDC1. To further dissect the roles of LCs and cDC1, we adoptively transferred LCs or cDC1 to the wound periphery (within 1.5 mm from the wound edge) of DT-exposed *Langerin*^DTR^ mice right after wounding. The number of transferred cells was estimated according to the normal number of LCs and cDC1 distributed in the wounding area [13]. LC repletion could reinstate the normal wound healing rate, while transfer of cDC1 had no obvious effect (Fig. 2B). Altogether, these data corroborate that LCs are required for a normal wound healing process.

### Escalated infiltration of neutrophils into the wounds in the absence of LCs

**Fig. 3:**
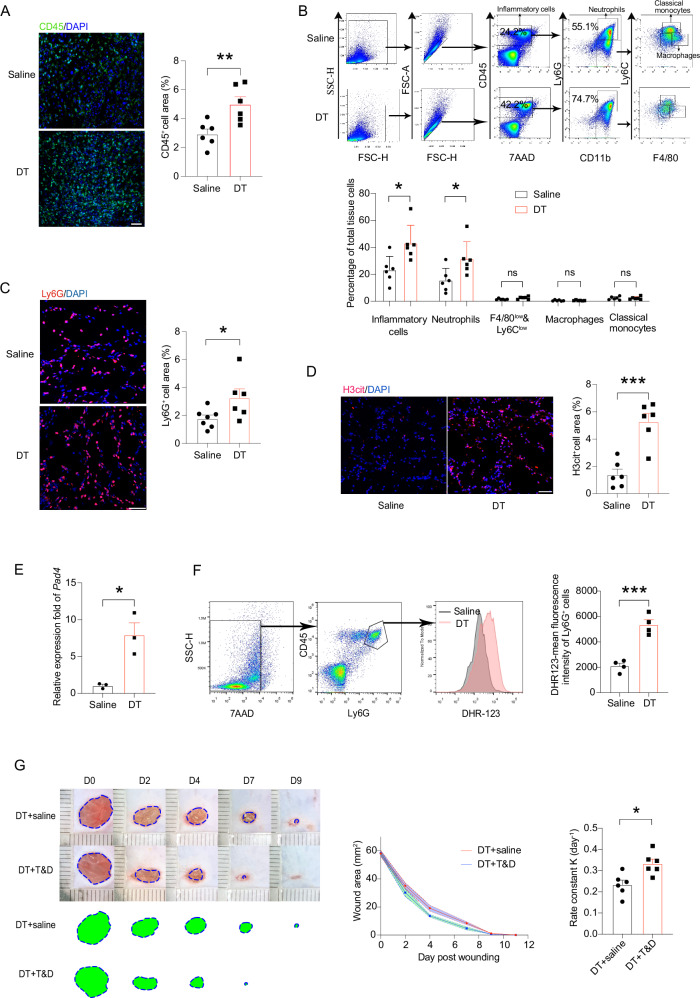
Increased number and activity of neutrophils in the wounds after LC removal. **A** Immunofluorescence staining showed inflammatory cells (CD45^+^) in the tissue of wound borders at day 2 after wounding. Scale bar: 50 µm. **B** Flow cytometry analysis of total inflammatory cells and myeloid cells in the day-2 wounds. **C** Immunohistostaining showed Ly6G^+^ neutrophils in the tissue of wound borders at day 2 after wounding. Scale bar: 50 μm. **D**, **E** H3cit^+^ cells (D) and expression of *Pad4* (E) in the wounds at day 2 after wounding. Scale bar: 50 μm. **F** ROS production was evaluated by the conversion of fluorescent DHR-123 via flow cytometry analysis of day-2 wounds. **G** LC-depleted mice were co-treated with (T&D) or without (SA) Tempol and DNase I. The representative wound images, kinetic statistics of wound area changes over time, and curve fitting rate were shown. The green area circled by dotted line represents the unhealed wound area. *P < 0.05, **P < 0.01, ***P < 0.001. ns, not significant by two-tailed unpaired *t* test. Data are depicted as mean ± SEM. Data are derived from at least 2 independent experiments.

Previous reports have established that the first 5 d post skin wounding is the acute inflammation stage marked by neutrophil influx [14]. This period corresponded to the period when the greatest differences were seen in LC-depleted versus LC-intact wound healing. We thus evaluated the degree of inflammation around the wounds on day 2 post wounding. In this study, wounds were collected by a 10-mm punch. Indeed, there was a greater infiltration of CD45^+^ inflammatory cells, in particular in the wound periphery, in the LC-depleted tissues relative to the LC-intact wound tissues, as demonstrated by immunohistostaining (Fig. 3A). Further analysis of whole wounds by flow cytometry unveiled that neutrophils were accountable for the majority of the increased inflammatory cells, whereas macrophages and inflammatory monocytes were underrepresented in both groups at this time point (Fig. 3B). Immunohistological staining of Ly6G also manifested an accumulation of neutrophils in the wound periphery and LC-depleted mice had more infiltrated neutrophils relative to the controls (Fig. 3C). To be noted, a recent study reported that local cDC1 could recruit neutrophil infiltration by producing VEGFα, an unconventional neutrophil chemoattractant, upon cutaneous bacterial infection [13], further excluding an off-target influence caused by a partial loss of cDC1 in the DT-treated mice. In accordance, we did not observe an altered expression of *Vegfa* transcripts in the LC-depleted wound tissues (Fig. S6).

Extruding neutrophil extracellular traps (NETs) and generating reactive oxygen species (ROS) are the hallmarks of neutrophil activation [15]. In alignment to the excessive influx of neutrophils in the LC-absent wound lesion, staining for citrullinated histone H3, a biomarker of NETs, exhibited exacerbated deposition of NETs in the wounds deficient of LCs (Fig. 3D). Elevated NETs production was also verified by the increased transcripts of peptidyl arginine deiminase 4 (PAD4) in the wound tissues (Fig. 3E). Moreover, staining with dehydrorhodamine 123, an indicator of ROS, displayed more ROS production by neutrophils in the LC-deficient wounds (Fig. 3F). Since a complete depletion of neutrophils will significantly retard wound closure [16], making this strategy unsuitable for investigating the influence of neutrophil over-influx. We next probed the hypothesis that the excessive accumulation of neutrophil-generated toxic molecules delays wound closure. To this end, we co-treated the LC-depleted mice with DNase I and tempol which could degrade NETs and scavenges ROS, respectively. Indeed, dampening the over-presence of NETs and ROS accelerated healing in the condition of LC depletion (Fig. 3G). Thus, an escalated neutrophil infiltration/activation in the skin was at least partially responsible for a slowed wound closure in the absence of LCs.

### Activation of CXCL5-CXCR1 pathway overdrives neutrophil infiltration into the wounds

**Fig. 4:**
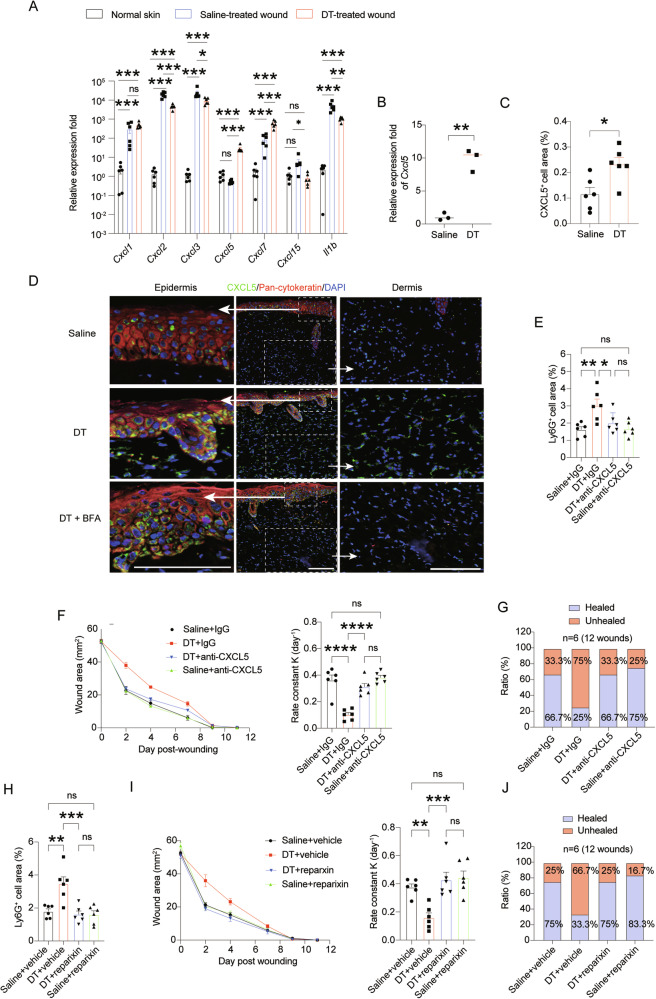
CXCL5-CXCR1 pathway are activated in the wounds absent of LCs, which overdrives neutrophil infiltration into the wounds. **A** The expression of a range of neutrophil chemokines at mRNA level in normal skin, normal wounds and LC-deficient wounds on hour 48 post wounding. **B** *Cxcl5* transcripts in normal wounds and LC-deleted wounds on hour 24 post wounding. **C** Immunohistostaining of CXCL5 expression in the wounds at hour 48 post wounding. **D** At hour 48 after wounding, LC-depleted mice (DT) were treated with or without brefeldin A (BFA). Four hours later, the wound tissues were harvested and were subject to CXCL5 and keratin immunohistostaining. Representative control wound tissue (Saline) without BFA treatment were also provided. Scale bar: 50 μm. **E**–**G** *Langerin*^DTR^ mice were pre-treated with saline or DT. Right after wounding, they were both separated to two groups receiving either an anti-CXCL5 antibody or an isotype antibody. **E** Immunohistostaining of Ly6G in the wounds were performed at hour 48 after wounding. **F** Kinetic statistics of wound area changes over time and curve fitting rate are shown. G. Frequencies of healed and unhealed wounds on day 9 after wounding. *n* = 6. **H**–**J**. *Langerin*^DTR^ mice were pre-treated with saline or DT. Right after wounding, they were both separated to two groups receiving either reparxin or vehicle. **H** Immunostaining of Ly6G in the wounds were performed at hour 48 after wounding. **I** Kinetic statistics of wound area changes over time and curve fitting rate are shown. **J** Frequencies of healed and unhealed wounds on day 9 after wounding. *n* = 6. *P < 0.05, **P < 0.01, ***P < 0.001, ****P < 0.0001, ns not significant by ordinary one-way ANOVA test (**A**, **E**, **F**, **H**, **I**) and two-tailed unpaired *t* test (**B**, **C**). Data are depicted as mean ± SEM. Data are derived from at least 2 independent experiments.

Next, we asked how the depletion of LCs affected the neutrophil influx into the skin lesion. To this end, we first collected the wound tissues and screened a plethora of chemotactic factors reported to mediate neutrophil recruitment at hour 48 post-wounding (Fig. 4A). While most of the tested factors were upregulated after wounding in both groups, the LC-deficient group had markedly higher expression of both *Cxcl5* (55.4 folds) and *Cxcl7* (9.7 folds) than their LC-intact littermates. We specifically focused on *Cxcl5* since it was the most pronouncedly altered. The overexpression of *Cxcl5* by LC-deficient mice could even be observed as early as 24 h post wounding (Fig. 4B). Immunohistostaining analysis confirmed more CXCL5^+^ cells in the LC-deficient wounds (Fig. 4C). The skin barrier is mainly composed of keratinocytes which are known to synthesize and release many chemokines when the barrier is breached [17]. To examine whether CXCL5 was mainly generated by the keratinocytes surrounding the wounds when LCs were depleted, we prepared sagittal sections in whole-mount sheets of backskins (epidermis+dermis+subcutaneous tissue) and co-stained CXCL5 with an anti-pan-cytokeratin antibody (Fig. 4D). The CXCL5 signals were enriched in the epidermal keratin^+^ keratinocytes cells, although there were some CXCL5 signals scattered in the dermis (Fig. 4D, upper panels). After depletion of LCs, CXCL5 expression were increased both in the epidermal and in the dermis (Fig. 4D, mid panels). Considering that CXCL5 is a releasable protein, to exclude the possibility that the CXCL5 signals on the keratinocytes were actually released by non-keratinocytes, we pre-treated the mice with brefeldin A which could block the secretion of intracellular proteins to the extracellular environment. In the presence of brefeldin A, only keratinocytes but no other cells in the whole-mount skin sections were positive in CXCL5 staining, confirming that the keratinocytes in the wound edge were the major source of CXCL5 in the absence of LCs (Fig. 4D, lower panels). Of note, DT-treatment alone to *Langerin*^DTR^ mice did not cause neutrophil infiltration or induce CXCL5 upregulation in the backskins without wounding (Figs. S7A and S7B), excluding an effect induced by LC apoptosis.

These results suggest that the exaggerated upregulation of CXCL5, or together with CXCL7, may be accountable to the neutrophil over-influx into the wounds in the absence of LCs. To interrogate this hypothesis, we treated LC-depleted mice with either a CXCL5-neutralizing antibody or an isotype IgG upon wounding. The wound tissues were examined 48 h later. Indeed, there was a marked reduction of neutrophils in the CXCL5-neutralized skin lesion relative to the isotype IgG-treated control littermates (Fig. 4E). When the wound closure rates were compared, the LC-deficient mice with CXCL5 abrogation could in a great extent restored the normalcy (Fig. 4F). In alignment, the frequencies of mice achieving a complete closure of wounds on Day 9 was boosted from 25% of the control mice treated with isotype antibody to 66.7% of those having CXCL5 blocked (Fig. 4G). The healing rate was actually comparable to those of the LC-intact controls (75% with anti-CXCL5 and 66.7% with isotype antibody).

We wondered why the over-production of CXCL5, or together with CXCL7, in the LC-ablated mice would lead to a marked over-influx of neutrophils, especially given the fact that other tested neutrophil chemotactic factors actually rose to a comparable or an even lesser degree in the wounds of LC-ablated mice relative to the controls. Intriguingly, among these chemokines, murine CXCL5 and CXCL7 are the only two factors ligating to both CXCR1 and CXCR2, while others selectively activate CXCR2 [18]. To probe the importance of CXCR1 in mediating the effect of neutrophil over-flooding in the wounds of LC ablation, we next treated mice with reparixin which has a 400-fold higher efficacy in inhibiting CXCR1 over CXCR2 [19]. Two days post-wounding, the LC-depleted mice which were treated with reparixin had a significant reduction of neutrophil infiltration into the skin wounds when compared to vehicle-treated LC-depleted mice (Fig. 4H). Moreover, application of reparixin improved wound healing, as demonstrated by a higher healing rate (Fig. 4I). As such, there was no difference in either neutrophil numbers on day-2 wounds or the healing rates between LC-present and LC-absent mice when reparixin was administered (Figs. 4H, I). Consistently, a complete closure of wounds on day 9 was boosted from 33.3% to 75% (Fig. 4J). Altogether, these data underscore an important role of the CXCL5-CXCR1 pathway in mediating neutrophil recruitment to the skin wounds in the absence of LCs. Blocking CXCL5-CXCR1 pathway could dampen neutrophil flooding and rescue wound healing of LC-ablated mice.

### LCs control the propagation of skin-symbiont bacteria in the wounds

**Fig. 5:**
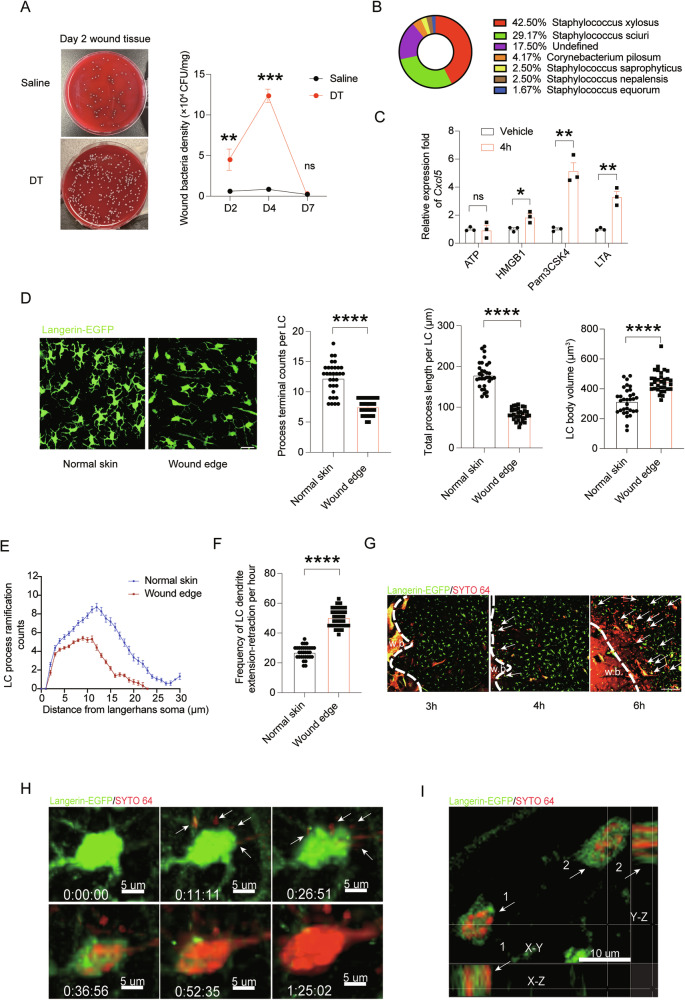
LCs control the invasion and propagation of skin-symbiont bacteria in wounds. **A** The number of bacteria in the wounds of LC-deficient mice (DT) and LC-intact mice (saline) at the indicated time after wounding. CFU: clone forming unit. **B** Mass spectrum results of wound bacteria indicate that the majority of the bacteria in the LC-deficient wounds were *Staphylococcus xylosus* and *Staphylococcus sciuri*. **C** Primary keratinocytes were stimulated with the indicated PAMPs or DAMPs molecules and their production of *Cxcl5* was measured. **D** Morphology analyses of LCs in Langerin-GFP reporter mice on hour 6 after wounding. Each dot indicates the quantification from one LC cell, 8 cells were selected randomly per mouse (*n* = 4) for statistical analysis. Scale bar: 20 μm. **E** Quantification of LC branch ramification complexity (illustration of Sholl analysis). **F** Two-photon microscopy analysis of LC dendrite changes in a 1-h observation period. The skin samples were derived from naïve *Langerin*^EGFP^ mice or the mice at hour 6 post wounding. Each dot represents the average frequency of all dendrites from one LC cell. 8 cells were selected randomly per mouse (*n* = 4) for statistical analysis. **G**–**I** SYTO 64-labeled bacteria were applied on top of the fresh wounds made on the backskins of *Langerin*^EGFP^ mice. **G** The interaction between bacteria (red) and LCs (green) was monitored in the indicated time points after wounding. Dotted line, border of wounds. Arrows, bacteria-phagocytosed LCs. Scale bar: 50 μm. **H** Time lapse of an LC and its associated bacteria at wound periphery 5 h post wounding. Arrows: free bacteria. **I** A representative two-photon image of wound epidermal sections show the bacteria (arrows, red) phagocytosed by LCs (green). Z-projections of 30 μm. *P < 0.05, **P < 0.01, ***P < 0.001, ****P < 0.0001, ns. not significant by ordinary one-way ANOVA test (**A**) and two-tailed unpaired *t* test (**C**, **D** and **F**). Data are depicted as mean ± SEM. Data are derived from at least 2 independent experiments.

Recently, Wasko et al. studied Langerin-DTA mice which had a constitutive loss of LCs and found that without LCs, angiogenesis was impaired during wound closure [20]. However, when we examined the wound beds of mice with induced LC loss on day 5 post puncture, a time point showing impaired angiogenesis by Wasko et al., angiogenesis appeared normal (Fig. S8). We posited that the increased production of CXCL5 by keratinocytes around the wounds of mice with induced LC loss (DT-treated *Langerin*^DTR^ mice) could be a response to an over-presence of bacteria in the wound area, since keratinocytes are sensitive to bacteria invasion by releasing chemokines for mobilizing immune cells, in particular neutrophils [7, 21]. To probe this possibility, we collected wound tissues in different time points post-wounding and homogenized them in a gentle speed for only degrading tissues but leaving cell uninjured. The homogenized tissues were then placed in blood agar plates for enumerating bacteria. To assure that mice were exposed to similar environment including commensal bacteria, DT- and the saline-treated *Langerin*^DTR^ littermates were housed for 1 week in the same cages before subject to the wounding model. Indeed, we observed markedly more bacteria present in the wound area of LC-depleted mice on Day 2~4 compared to LC-intact littermates (Fig. 5A). However, bacteria number in the LC-depleted wounds significantly decreased on Day 7, possibly due to a containment by the over-flooded neutrophils. To examine the identities of the bacteria, we picked a total of 120 bacteria colonies from the blood agar plates of the Day 2 wound tissues originated from 6 LC-deficient mice for mass spectrometry analysis. It exhibited that the majority of the bacteria in the LC-deficient wounds were *Staphylococcus xylosus* and *Staphylococcus sciuri* (Fig. 5B), both of which belong to coagulase negative *staphylococcus* and are skin-symbiont bacteria colonizing in normal skins [22]. This finding suggests that LCs are critical in containing the propagation of skin-symbiont bacteria in the wounds. Supporting the idea that staphylococcus outgrowth was a dominant factor over locally released danger-associated molecular patterns (DAMPs) that irritated the overproduction of CXCL5 from keratinocytes, we found that lipoteichoic acid (LTA), a major constituent of the cell wall of Gram-positive bacteria, as well as the triacylated lipopeptide (Pam_3_CSK_4_), a synthetic ligand of TLR1/TLR2, was more efficient in upregulating *Cxcl5* expression than ATP and HMGB1, two common DAMPs associated with wound injuries, in primary murine keratinocytes (Fig. 5C).

To understand how LCs react to the skin-colonizing bacteria after wounding, we employed the *Langerin*^EGFP^ reporter mice and punched their backskins by a 23 gauge needle. Six hours later, we examined the epidermis in the wound edge (within a distance of 500 μm to the site of puncture) by confocal microscopy. LCs bordering the wound edge displayed profound changes in morphology: they retracted their dendrites and became rounded, with a significant increase in their soma volume and a notable decrease in both dendrite number and length on per cell basis (Fig. 5D). Reduction of the degree of their ramification after wounding was also observed by Sholl analysis (Fig. 5E). These morphological changes with enlarged cell bodies and reduced dendrite complexity are reminiscent of the alterations occurring during microglia activation in the central nervous system [23], indicative of LC activation. To further evaluate the motion of LCs, we set up two-photon microscopy to analyze the temporal behavior of LCs at wound edge. Although the bodies of LCs remained immobile in the epidermis after wounding, their dendrites displayed accelerated extension-and-retraction movements (Fig. 5F), appearing to be actively sampling environmental substances. Some cells could even de novo protrude dendrites with a wrapping maneuver (Movies S1), indicative of phagocytosing large particles. To monitor potential interaction between bacteria and LCs, we topically applied 10^8^ colony-forming units (CFUs) of live fluorescently labeled staphylococcus (*S. xylosus* and *S. sciuri* in a 1:1 mixture) to needle-punched wounds. At the hour-3 wounds, most labeled bacteria still stayed in the wound front (Fig. 5G). As early as hour 4, bacteria infiltration into the wounds was observed and some bacteria were already associated with LCs (Fig. 5G and Movie S2). By 6 h, most of the infiltrating bacteria had been phagocytosed by LCs (Fig. 5G), strongly suggesting that the LCs are the major cells in clearing off bacteria in the beginning stage of bacteria infiltration. One LC could encounter and intake multiple bacteria in situ (Fig. 5H) and the intracellular bacteria were verified by XZ and YZ sections (Fig. 5I). Altogether, these data indicate that in the absence of LCs, skin-symbiont bacteria have a greater chance to invade and propagate inside the wounds, which would eventually lead to an exaggerated neutrophil influx by instigated keratinocytes.

### Anti-bacteria treatment can rescue the slowed wound healing process caused by LC loss

**Fig. 6:**
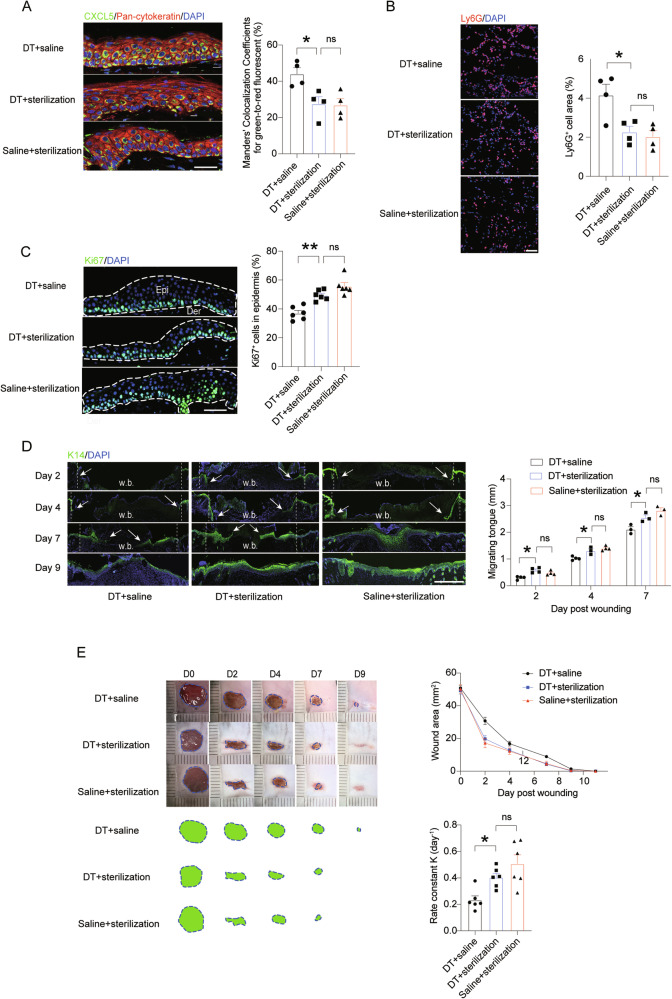
Anti-bacteria treatment can rescue the slowed wound healing caused by LC loss. *Langerin*^DTR^ mice were pre-treated with saline or DT. Right after wounding, they received daily topical application of benzalkonium chloride or saline. For **A**–**C**, Scale bar: 50 μm. **A** CXCL5 expression in the epidermis on day 2 after wounding. Scale bar: 50 μm. **B** Neutrophil (Ly6G) infiltration in the wounds on day 2 after wounding. Scale bar: 50 μm. **C** Ki67^+^ proliferating cells in the epidermis on day 2 after wounding. The white dotted area represents the epidermis at the edge of the wound. Epi: epidermis; Der: dermis. **D** Migration of epidermal progenitor cells (K14^+^) was measured in the indicated time points after wounding. The dotted lines indicate the initial wound edge, and arrows mark wound tongues. w.b., wound bed. Scale bar: 1 mm. **E** Representative wound images, kinetic statistics of wound area changes over time and curve fitting rate are shown. *P < 0.05, **P < 0.01, ns not significant by ordinary one-way ANOVA test (**A**, **B**, **C** and **E**) or two-way ANOVA with multiple comparisons (**D**). Data are depicted as mean ± SEM. Data are derived from at least 2 independent experiments.

To interrogate whether bacteria overgrowth was a result from a delayed wound closure, we counted bacteria number in the wounds of day 1 when there was no difference in wound size between LC-depleted and LC-intact mice (Fig. S9). It displayed that the LC-depleted wounds already had remarkably more bacteria than the controls on day 1 post-wounding, suggesting that a delayed wound closure was a result but not a cause of bacteria over-presence in the LC-deficient wounds. To substantiate the hypothesis that bacteria overgrowth in the early wounds of LC-ablated mice was responsible for a slowed wound healing, we topically applied benzalkonium chloride, an antimicrobial agent widely used to prevent skin infection, onto the wound area. The bacterial load in the wounds of DT-injected mice significantly decreased following disinfection (Fig. S10). Compared to the LC-depleted mice topically applied with saline, daily benzalkonium treatment significantly reduced CXCL5 production and neutrophil influx, and at the same time increased the number of proliferating epidermal basal cells on Day 2 post wounding (Fig. 6A–C). More importantly, with daily skin sterilization, extension of K14^+^ epithelial tongue was accelerated (Fig. 6D), accompanied with a greater rate of wound closure (Fig. 6E). Of note, there was no difference between sterilized LC-deficient and LC-present mice in CXCL5 level, neutrophil influx, K14^+^ cell migration and wound healing rate, strongly suggesting that a complete rescue to LC-deficient mice could be achieved via suppressing commensal bacteria overgrowth, which also implies that impaired bacteria-containing but not other effects of LC loss was attributable to a delayed wound healing.

Altogether, these studies substantiate a pivotal role of LCs in containing symbiotic bacteria propagation in the area of skin wounding, which is important for a proper degree of inflammation and is overall beneficial to the followed wound healing process.

## Discussion

LCs are located in the epidermis and are the outermost phagocytes to encounter pathogens at the skin surface. Skin is colonized with a plethora of commensal bacteria of which staphylococcus is the most abundant species [24]. It has been shown that the number of epidermal LCs is reduced in certain skin conditions such as aged skin [25], UV-irradiated skin [26], and sarcoidosis [27]. Those circumstances commonly show the signs of delayed barrier recovery, with impaired epidermal barrier function [28–30]. However, the underpinning mechanisms were not fully understood. In the current study, we demonstrated that LCs were required for warding off bacteria invasion at the early stage of skin wounds. Without LCs, *S. xylosus* and *S. sciuri* which are generally not pathogenic commensals were overrepresented in the wounding area. In such an occasion, keratinocytes surrounding the wounds would respond to the excessive bacteria by an active production of CXCL5 and CXCL7 in addition to other neutrophil-recruiting chemokines. Being uniquely mediated through CXCR1 locally increased CXCL5 and CXCL7 attracted significantly more neutrophils to the wounds at the early stage. The over-presence of activated neutrophils in the absence of LCs, possibly together with the aggravated invasion of bacteria, was detrimental re-epithelialization. As such, wound healing was markedly slowed in the early stage. Topical application of benzalkonium chloride, an antimicrobial agent, could fully rescue the healing process in the wounds abrogated of LCs. These observations underscore an indispensable role of LCs as effective guardians that preclude both bacteria invasion and possible damages inflicted by over-inflammation. Moreover, this study highlights a particular efficient keratinocyte-neutrophil communication through CXCL5/CXCL7-CXCR1 pathway. In condition of severe breaching of staphylococcus or possibly other bacteria as well, keratinocyte started to generate abundant CXCL5/CXCL7 which could improve the efficiency of neutrophil entry for containing pathogen propagation.

Without LC-specific gene ablation, the precise role of LCs in modulating the CXCL5-CXCR1 axis remains incompletely defined. Our study shows LCs contribute to, but are not the sole modulators of, CXCL5-CXCR1 axis-related processes during wound healing, which acknowledges the potential roles of other cell types, such as keratinocytes, in producing chemokines and regulating neutrophil activity. Further studies would be needed to dissect the specific contributions of LCs versus other skin-resident cells.

In our study, it appears that the phenotypic differences between the saline and DT groups in BM recipients (Fig. 2A) are attenuated compared to normal mice (Fig. 1D). This change may indeed be associated with the bone marrow transplantation procedure itself. Following bone marrow transplantation, mice undergo a series of systemic changes, most notably the reconstruction of the hematopoietic immune system. Despite employing controls, numerous factors inherent to the transplantation process may influence phenotypic differences: (1) Age discrepancy: Although all mice commenced the experiment at 8 weeks of age, recipients of bone marrow transplantation require an additional 8-week period post-transplantation to ensure successful and complete immune system reconstitution. Variations in the age of mice may result in differences in the rate and process of wound repair for the same injury. (2) Immunological alterations induced by bone marrow transplantation encompass changes in the hematopoietic immune system and alterations in cutaneous appendages (for example, resulting in hair graying), which may obscure or interfere with the phenotype of LCs in wound healing.

Resonating with our observation, a previous study showed that intradermal administration of recombinant GM-CSF could recruit LCs, improve keratinocyte growth and enhance wound healing [31]. Here, we provided a mechanism for the beneficial role of LCs in wound healing. Our findings also echo with a recent study which demonstrated that more than antigen presentation, LCs had a bactericidal activity [32]. Actually, the role of LCs in wound healing has been examined in different transgenic mouse models with mixed conclusions [11, 33]. One of the reasons for the controversy is that these models made use of transgenic mice depending on the promoter of *Cd207* (langerin) or *Itgax* (CD11c) both of which were shared between LCs and DCs so that a definite function of LCs could not be elucidated. In this study, we revisited the role of LCs in wound healing by using a mouse model with inducible LC-depletion. In particular, we excluded a mixed effect of cDC1 by bone marrow transplantation and adoptive transfer experiments. While the method of using in vitro differentiated cDC1 for in vivo transplantation has certain limitations, it can still provide valuable insights into the functional characteristics of cDC1. This approach is widely utilized in the field, underscoring its relevance despite the inherent challenges. In particular, a recent study also employing the *Langerin*^DTR^ mice made a contradictory observation which showed that loss of langerin^+^ cells would accelerate wound healing [11]. After careful evaluation, we found that the authors used a dose of DT, i.e., 1 μg, which resulted in variety of off-target effects including a complete depletion of dermal cDC1, local inflammation and tissue cell apoptosis, profoundly complicating data interpretation. In the current study, a tenfold lower dose of DT, i.e., 100 ng, was used, which had milder effect on cDC1 and more importantly, did not cause apparent inflammation and tissue apoptosis. Here, we excluded the off-target effect of cDC1 removal via additional experiments including bone marrow transplantation and cell adoptive transfer, substantiating a beneficial role in containing an exaggerated neutrophil infiltration and in the processing of re-epithelialization.

A recent study also interrogated the role of LCs in wound healing by using Langerin-DTA mice [20]. In these mice, active subunit of diphtheria toxin is expressed in LC, resulting in a constitutive and durable absence of LCs. Consistent with our findings, this study found a beneficial role of LCs in wound healing. However, the author attributed these effects to a supportive role of LCs in angiogenesis. In contrast, we did not find a significant difference in angiogenesis in our inducible LC-removal system. Considering that LCs may have roles in the skin development and have effects on skin composition, our inducible LC-deficiency model is different from the model with a constitutive LC loss, which was supported by an observation that Langerin-DTA mice had a loss of epidermal sensory afferent nerves [34, 35]. LCs were also shown to stimulate keratinocytes by providing EGF in healing from ultraviolet radiation [36]. Thus, it is not surprising to see that these resident innate immune cells in the epidermis have multiple influences on the surrounding cells in both steady state and in the healing processes of various nature of wounds.

Future research may focus on investigating the regulatory mechanisms of LC activity, exploring the optimal density needed for effective healing, and examining their interactions with other immune cells. Additionally, studies should evaluate therapeutic approaches to modulate LC function, conduct clinical trials on LC-targeted therapies for chronic wounds, and develop biomarkers to personalize treatment strategies. These efforts could enhance our understanding of LCs’ roles and lead to innovative therapies for improved wound healing outcomes.

## Methods

### Mice

B6.129S2-Cd207^tm3(DTR/EGFP)Mal/J^ (*Langerin*^DTR^, Stock#: 016940) and B6.129S2-Cd207^tm2.1Mal/J^ (*Langerin*^EGFP^, Stock#:016939) mice were purchased from the Jackson Laboratory. Normal C57BL/6 mice were provided by Shanghai Research Center for Model Organisms. All mice used in this study without specific explanation were 8~9-week-old males. Mice were housed in a standard animal facility, with a 12-h light/dark cycle, in specific-pathogen-free environment.

### Skin wounding models

Mice during telogen phase of hair cycling were wounded under anesthetization. Briefly, dorsal hair was shaved and depilated with depilatory cream. After skin was swabbed with 75% ethanol, two symmetrical full-thickness skin defects were made on their back with a biopsy punch (8 mm in diameter). For silicone splinted wound model, a silicone splint (9.5 mm in inner diameter, 14.5 mm in outer diameter and 0.8 mm in thickness) was affixed around full-thickness wounds [37]. A vaseline gauze was bandaged to the surface of the splinted wound during the whole wound healing procedure. Utilize a 10 mm biopsy punch to collect tissue samples from the wound site, ensuring uniformity in tissue size for accurate analysis. For an acute micro-wound model, a 23G needle was used to make a full-thickness puncture at the back skin. To calculate the wound healing rate, a one-phase decay model (Graphpad prism) is applied according to the reference [12].

### Reagents

Unless specified, diptheria toxin (Listbio) was i.p. given at a dose of 100 ng/mouse every 5 days for 11 days. A CXCL5 neutralizing antibody (MAB433, RD) was i.p. injected at the dose of 50 μg/mouse right after wounding, and the control group was given a same dose of isotype antibody (E-AB-F098430, Elabscience). To block CXCR1, mice were i.p. injected with reparixin (S8640, Selleck) at the dose of 30 mg/kg/48 h for 9 days. Tempol (Selleck) and DNase I (Sigma) were injected i.p. with the dose of 50 μg/each/12 h and 100 mg/kg/24 h for 9 days, respectively. In some experiments, the wound surface was applied with 0.1% benzalkonium chloride (Lustre Pharmaceutical Lab) every day until wound closure was achieved.

### Cell collection and adoptive transfer

To collect cDC1, total bone marrow cells were cultured in RPMI 1640 complete medium (containing 10% fetal calf serum, 1% penicillin/streptomycin) supplemented with 50 μΜ 2-mercaptoethanol, 200 ng/ml recombinant FLT3 (PeproTech) and 5 ng/ml recombinant GM-CSF (PeproTech). Medium was changed on Day 5 and Day 9. The non-adherent cells were collected on day 15 followed by flow cytometry sorting to purify cDC1 (CD45^+^CD11b^–^MHC-II^+^CD103^+^XCR1^+^). To collect primary LCs, epidermis was separated from dermis according to an established protocol [38] with slight modifications. Briefly, dorsal skin was shaved and the subcutaneous fat was removed, followed by digestion in a digestive solution containing 100 μ/ml DNase I and 5 mg/ml dispase at 37 °C for 1 h with the dermal side down. With the aid of forceps, the epidermis was peeled from the underlying dermis and subjected to further digestion in a complete culture medium supplemented with 100 μg/ml of DNase and 0.05% trypsin within a shaking water bath at 37 °C for 20 min, followed by vigorous pipetting and passing through a 70 µm filter to obtain a single-cell suspension. LC was purified by flow cytometry sorting (CD45^+^CD11c^+^CD207^+^). 5 × 10^5^ LC and 5 × 10^4^ cDC1 were individually injected to the wound bed and wound edge immediately after trauma.

### Bone marrow transplantation

Bone marrow was obtained from 8-week-old donors. Nucleated cells were counted, and the cells were resuspended at a concentration of 2 × 10^7^/ml. Recipient mice were irradiated with 950 rads and then were immediately i.v. reconstituted with 2 × 10^6^ bone marrow cells.

### Cell Isolation and flow cytometry

The separation of epidermal cells is as described above. After removing the epidermal layer, the remaining dermal tissue was minced, digested with 0.2% type I collagenase at 37 °C for 30 min, and was filtered through a 70 µm filter. The preparation method for full-thickness skin is the same. Samples were analyzed with a 3-laser flow cytometer (Agilent Novocyte) and data were processed with FlowJo (v10.1). LCs and cDC1 were purified by cell sorting with a BD SORP ARIA II.

The following fluorophore-conjugated antibodies were purchased from BioLegend or eBioscience and used for flow cytometry analyses: anti–CD45 (30-F11), anti–CD11c (N418), anti–CD207 (4C7), anti–CD11b (M1/70), anti-Ly-6G (1A8), anti–Ly6C (HK1.4), anti–F4/80 (BM8), anti–I-A/I-E (M5/114.15.2), anti-XCR1 (ZET) and anti–CD103 (2E7) were used. Dead cells were excluded with 7-amino-actinomycin D Viability Staining Solution (BioLegend).

### Quantitative real-time PCR

Wound tissue and cell whole RNA was isolated by using the ESscience RNA-Quick Purification Kit (RN001 and RN002, respectively). Hifair III 1st Strand cDNA Synthesis SuperMix for qRT-PCR (Yeasen Biotechnology, shanghai) was used for cDNA synthesis. Hieff qPCR SYBR Green Master Mix (Yeasen Biotechnology, shanghai) were used for signal generation detected by CFX96 Touch Real-Time PCR Detection System (Bio-Rad). Relative expression levels were calculated as transcript levels of target genes relative to housekeeping gene *Gadph* (primers’ details see Table 1).

**Table 1:** Primers’ details.

Gene name	Species	Forward primers	Reverse primers
*Gapdh*	Mouse	GGTGAAGGTCGGTGTGAACG	CTCGCTCCTGGAAGATGGTG
*Cxcl1*	Mouse	CTGGGATTCACCTCAAGAACATC	CAGGGTCAAGGCAAGCCTC
*Cxcl2*	Mouse	CCAACCACCAGGCTACAGG	GCGTCACACTCAAGCTCTG
*Cxcl3*	Mouse	AGGCCCCAGGCTTCAGATAAT	AATGCAGGTCCTTCATCATGGT
*Cxcl5*	Mouse	TGCGTTGTGTTTGCTTAACCG	CTTCCACCGTAGGGCACTG
*Cxcl7*	Mouse	CTCAGACCTACATCGTCCTGC	GTGGCTATCACTTCCACATCAG
*Cxcl15*	Mouse	CAAGGCTGGTCCATGCTCC	TGCTATCACTTCCTTTCTGTTGC
*Il1b*	Mouse	TTCAGGCAGGCAGTATCACTC	GAAGGTCCACGGGAAAGACAC
*Il6*	Mouse	TAGTCCTTCCTACCCCAATTTCC	TTGGTCCTTAGCCACTCCTTC
*Tnfa*	Mouse	CCCTCACACTCAGATCATCTTCT	GCTACGACGTGGGCTACAG
*Padi4*	Mouse	TCTGCTCCTAAGGGCTACACA	GTCCAGAGGCCATTTGGAGG
*Vegfa*	Mouse	GCACATAGAGAGAATGAGCTTCC	CTCCGCTCTGAACAAGGCT

### Immunofluorescence

For immunostaining on sections, wound or skin tissues was embedded in OCT or Paraffin. For frozen samples, sections of 10 µm were used and fixed in 4% paraformaldehyde for 10 min at room temperature. For paraffin sections (5 µm), slides were placed in sodium citrate buffer (pH = 6.0) to repair the antigen after deparaffinization and hydration. Tissues were then permeabilized with 0.1% Triton X100 for 10 min and incubated in blocking buffer (5% BSA, 5% donkey serum, 0.1% Triton, 0.1% gelatin in PBS) for 1 h at room temperature. The following primary antibodies were used: anti-K14 (Rabit, 1:200, ab119695, Abcam), anti-Ki67 (rabbit, 1:200, GB111141, service), anti-CD31 (Rat 1:200, 102501, Biolegend), anti-CD45 (Rat, 1:100, 14-0451-82, eBioscience), anti-Ly6G (Rat, 1:200, 551459, BD), anti-H3Cit (rabbit, 1:300, ab5103, Abcam), anti- CXCL5 (1:100, BS2549R, Bioss) and Mouse pan-cytokeratin (1:200, ab86734, Abcam). Sections were then rinsed and incubated with secondary antibodies in blocking buffer for 1 h at room temperature. Apoptotic cells were detected by Fluorescein Tunel Cell Apoptosis Detection Kit (G1501-50T, Servicebio). After nuclei staining with 4′6′-diamidino-2-phenylindole (DAPI), Images were acquired with confocal microscope (Carl Zeiss LSM 900), and analyzed with the Image J software.

### Analysis of the motility and morphology of LC

*Langerin*^EGFP^ mice were randomly divided into micro-wound group and control group. After the mice were anesthetized and depilated, a micro-wound was made by the method talked above. The mice were fixed under a two-photon microscope (FVMPE-RS, Olympus) to observe the morphology of LCs in the skin around the wound. For morphological evaluation, Sholl analysis was used to evaluate dendritic structure and branching complexity by quantifying the number of intersecting branch points on concentric circles with a specific distance from the cell body. Sholl analysis parameters are as follows: the starting point of concentric radius is set at the midpoint of the longest axis of the soma, the starting radius is 1 μm with increased radii (1 μm intervals), end at 27 μm radius. In general, in a given experiment, an average of 8 LCs per mouse were quantified for sholl analysis, dendritic length, branch number and body volume. For evaluating the interactions between LCs and bacteria, 5 μl PBS containing 1 × 10^8^ live staphylococci labeled with dye SYTO 64 was applied to the wound. The morphology and movement of LCs in the skin around the wound were observed under a two-photon microscope 3 h later. The two-photon microscope is equipped with modules that can perform four-dimensional analysis (*x*, *y*, *z*, *t*). LCs and bacteria were observed and recorded under the followed conditions: 20× Objective lens, 25~30 μm recording range in Z-axis, 1.5 μm *Z*-axis increment, record every 40 s for 1–2 h with a resolution of 512 × 512 pixels. The laser was tuned to 920-nm excitation and used in all relative experiments. Images and videos were processed and analyzed with imaris (bitplane, Switzerland) software.

### Culture and stimulation of mouse primary keratinocytes

The whole skin of newborn mice (1–3 days after birth) was peeled off and placed in the digestive solution containing 4 mg/ml dispase and 1% penicillin/streptomycin, and then digested at 4 °C for 12–14 h. The epidermis was peeled off with tweezers, placed in 0.25% trypsin and 2 mM EDTA, and digested at 37 °C for 30 min. After filtering with a 70 µm filter screen, cells were spinned at 300 g for 5 min at 4 °C and then resuspended in a keratinocyte complete culture medium containing Medium 154CF, Kit (M154CF500, Gibco) and 1% penicillin/streptomycin. Medium was changed once every 3–5 days. Primary keratinocytes of passages 1–3 were stimulated by Pam3CSK4 (500 ng/ml), LTA (10 μg/ml), HMGB1 (100 ng/ml) or ATP (100 μM). Total RNAs of cells were extracted after 4 hours of stimulation for subsequent expression analysis.

### Quantification of wound bacteria

After weighing, the removed wound tissue was minced and put into a sterile EP tube and fully homogenized in 500 μl sterile water. In tittering, 30 μl diluent was added to the blood plate and smeared evenly. The number of bacterial clones was calculated after incubation in a 37 °C incubator for 24 h. Bacterial clones were picked and analyzed by mass spectrometry (MicroTyper).

### Statistical analyses

Statistical analysis was performed with Prism 6.0 (GraphPad). Data are presented as mean ± SEM. Unpaired Student’s *t* tests, One-way ANOVA or Two-way ANOVA with post hoc tests were used. All statistical tests were two-tailed, and *P* values of <0.05 were considered significant.

## Supplementary information

Movies S1

Movies S2

Movie legends

sFigure and legends

## Data Availability

All data and materials generated in this study are available from the corresponding author upon reasonable request.
