# Supplementary material for: Absence of Langerhans cells resulted in over-influx of neutrophils and increased bacterial burden in skin wounds
Source: Cell Death Dis. 2024 Oct 19;15(10):760. doi: 10.1038/s41419-024-07143-1 (PMC11489468; doi:10.1038/s41419-024-07143-1)
Supplement: Supplementary file 3 — Movie legends [file 41419_2024_7143_MOESM3_ESM.docx]

**Movies S1. LCs at wound edge would protrude dendrites with a wrapping maneuver.** Time lapse video of epidermis at the back of a *Langerin*^EGFP^ mouse starting from 5 hr post needle pucture, taken by a time-lapse two-photon microscopy. Frames were collected at the frequency of every 40 second. The display rate is 8 frames/second. Green: LCs. Arrows, protruding dendrites.

**Movies S2. Infiltrating bacteria were uptaken by the residing LCs in the epidermis.** a Needle-punched wounds were made on *Langerin*^EGFP^ mice, and SYTO 64-labeled staphylococcus was topically applied. Five hours later, the wounds were observed with a two-photon microscopy. Time lapse video of wound edge taken by a two-photon microscopy is shown. A LC (green) was intaking the encountered bacteria (red) and the bacteria accumulated in the LC. Frames were collected at the frequency of every 40 second. The display rate is 7 frames/second.
