# Supplementary material for: Absence of Langerhans cells resulted in over-influx of neutrophils and increased bacterial burden in skin wounds
Source: Cell Death Dis. 2024 Oct 19;15(10):760. doi: 10.1038/s41419-024-07143-1 (PMC11489468; doi:10.1038/s41419-024-07143-1)
Supplement: Supplementary file 4 — sFigure and legends [file 41419_2024_7143_MOESM4_ESM.pdf]

Fig. S1

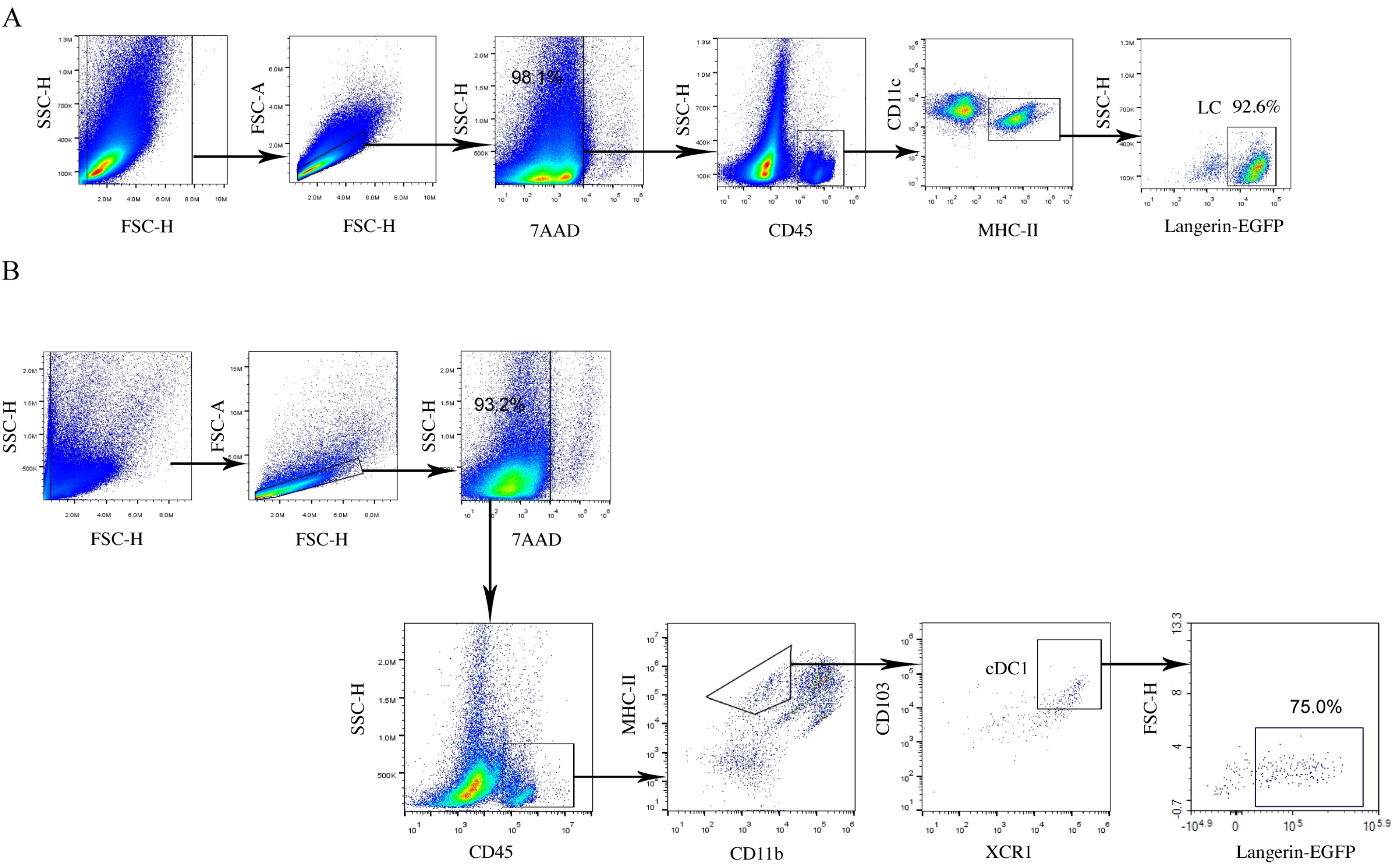

Figure S1. Flow cytometry analysis of the langerin expression in LCs and dermal cDC1.

The gating strategies and representative dot plots for analyzing CD207 (langerin) expression in epidermal LCs (**A**) and dermal cDC1 (**B**) by using the *Langerin*^{EGFP} reporter mice.

Fig. S2

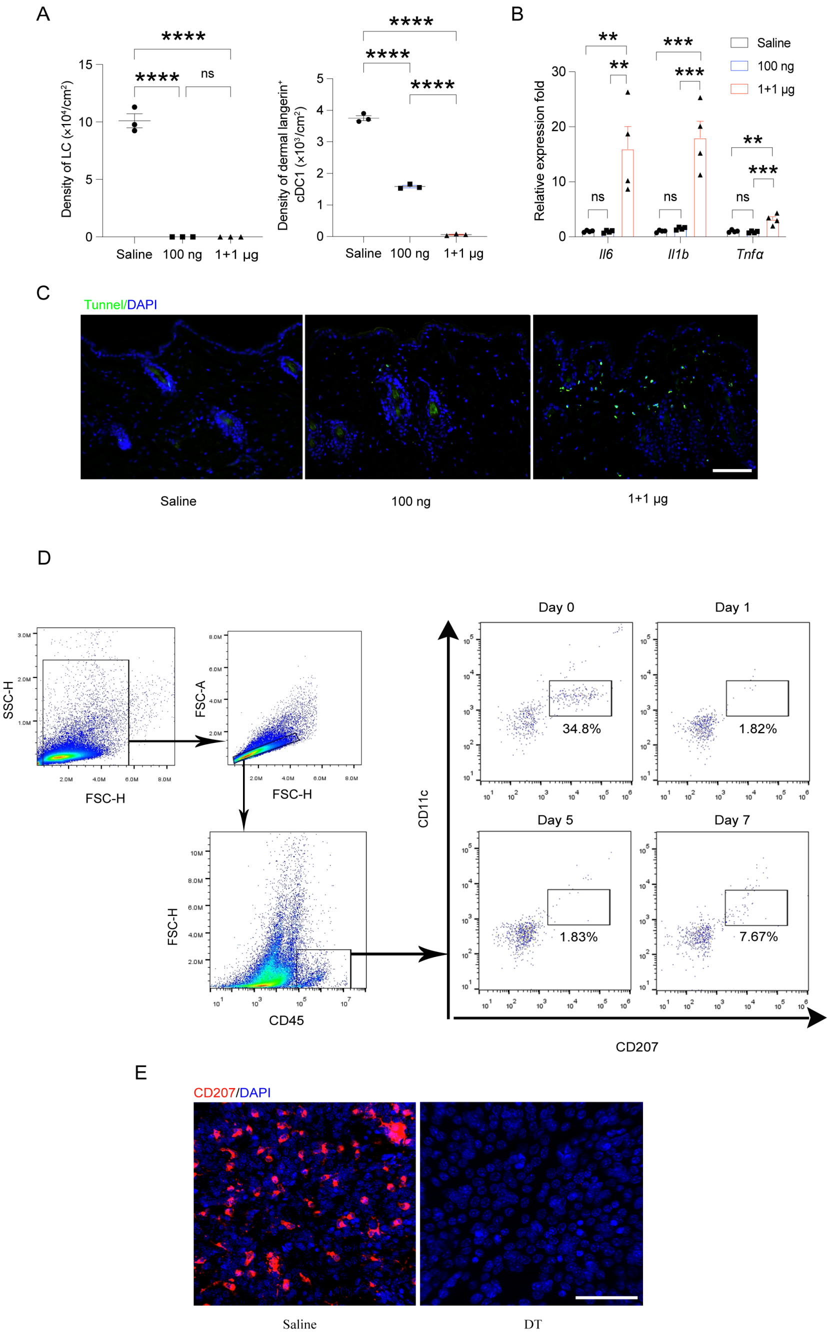

Figure S2. Efficiency of DT in removing LCs and dermal cDC1 in *Langerin*^{DTR} mice. A. The efficiency in removal of epidermal LCs and dermal cDC1 24 hr post 100 ng or 1 μg DT administration was evaluated by flow cytometry analysis. **B.** Expression of the indicated cytokines in the backskin was measured by RT-PCR. **C**. TUNEL staining shows apoptotic cells in the dermis at hour 24 post the indicated treatment. Scale bar: 50 μm. **D.** Flow cytometry analysis of epidermal LCs at the indicated time point after 100 ng DT treatment. **E.** CD207 staining shows LCs depletion in the epidermis at hour 24 post DT treatment. Statistical tests: ordinary one-way ANOVA with multiple comparisons. ** P<0.01, ***P<0.001, **** P<0.0001, ns, not significant. Data are depicted as mean±SEM.

Fig. S3

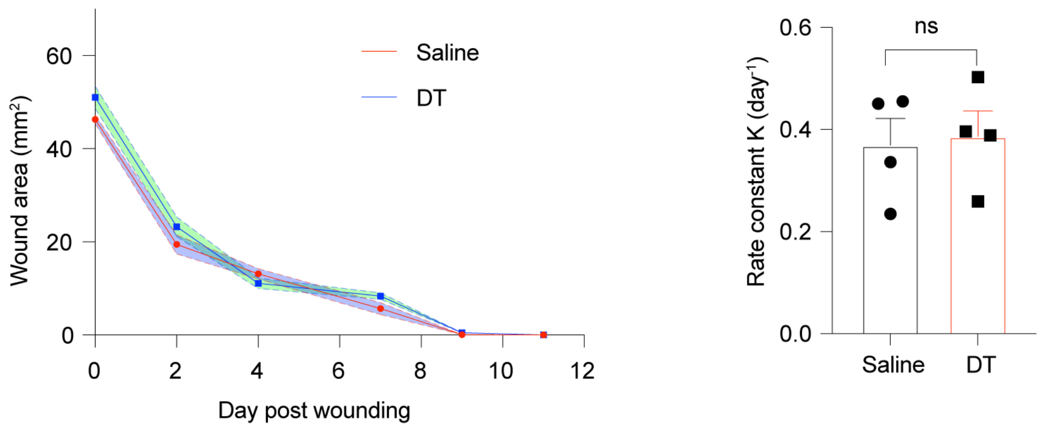

Figure S3. DT did not alter the wound healing process in C57BL/6 mice. Eight-week-old C57BL/6 mice were subject to dorsal skin full-thickness wounding. Kinetic statistics of wound area changes over time (left) and curve fitting rate calculated based on the unhealed wound area over the observation period (right) are shown. ns. not significant. Data are depicted as mean±SEM.

Fig. S4

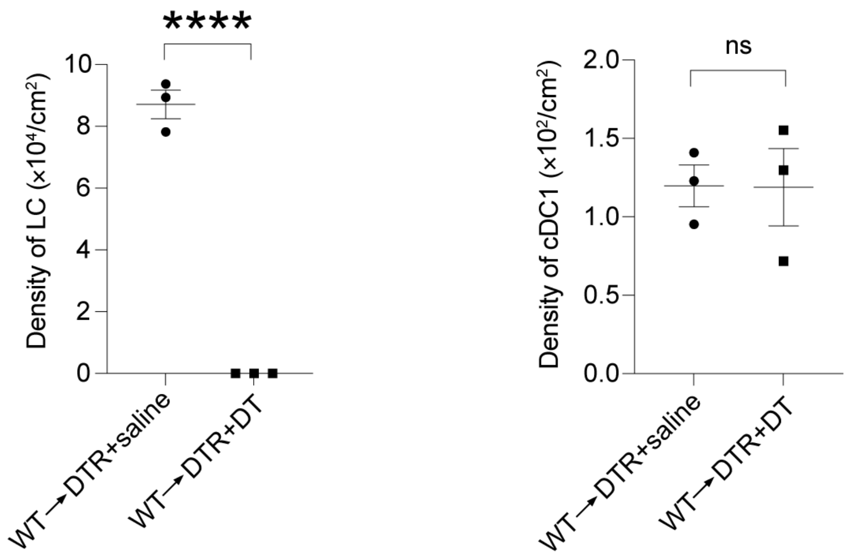

Figure S4. Flow cytometry analysis of epidermal LCs (left) and dermal cDC1 (right) density of WT BM-reconstituted *Langerin*^{DTR} mice after 100 ng DT treatment. ****: P<0.0001, ns: not significant. Data are depicted as mean±SEM.

Fig. S5

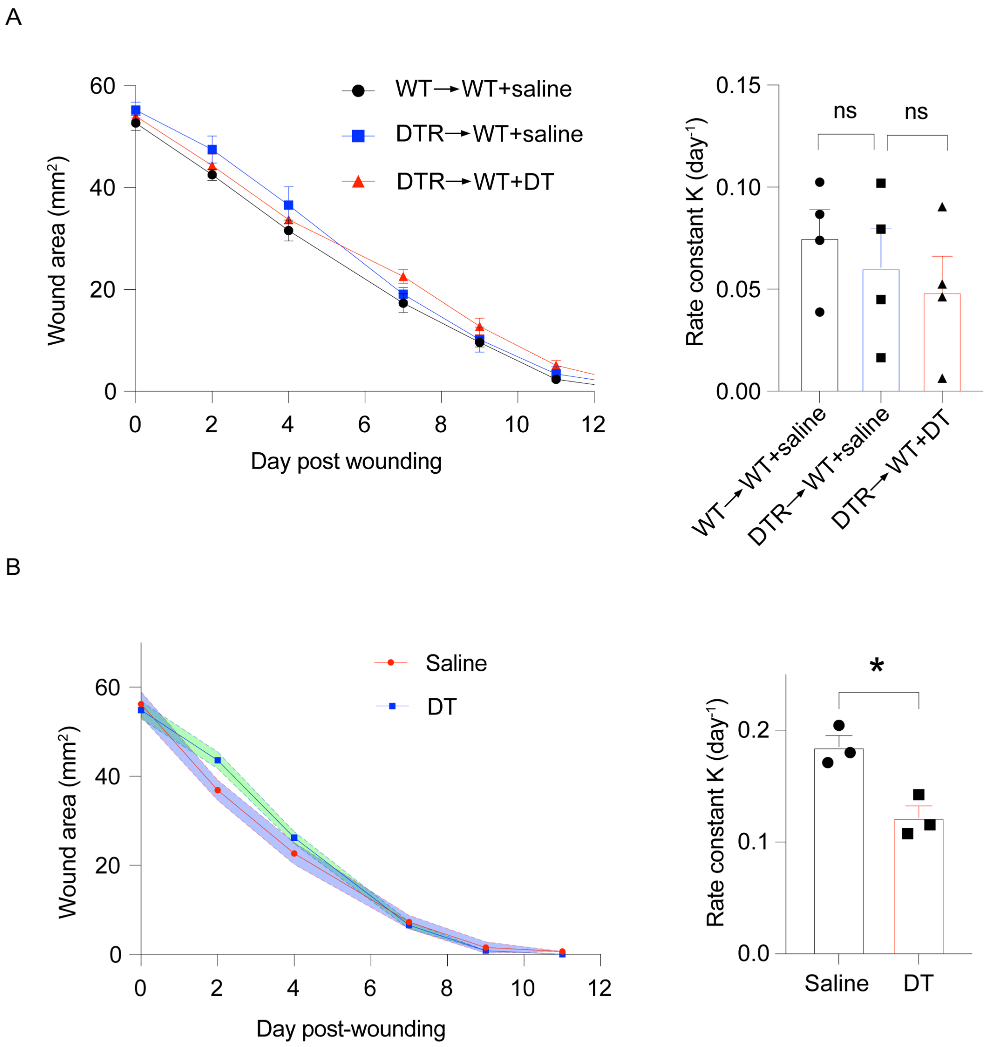

Figure S5. Depleting LC, not langerin^{+} cDC1 showed delayed wound healing rate. A. WT mice received either WT BM or *Langerin*^{DTR} mouse BM. The kinetic statistics of wound area changes over time, and curve fitting rate in a silicone affixed wound mode were shown. **B.** 4 weeks after *Langerin*^{DTR} mice injected with saline or DT, wound with DT injection was delayed comparing with saline. *: P<0.05, ns, not significant. Data are depicted as mean±SEM.

Fig. S6

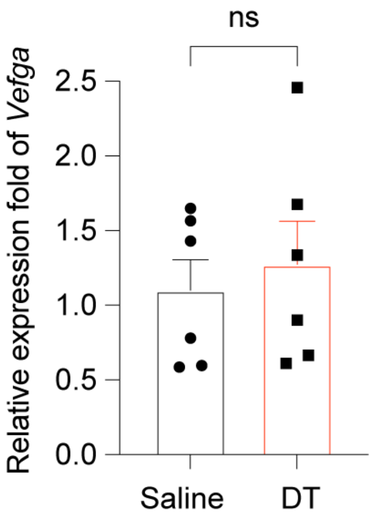

Figure S6. There was no significant difference in the expression level of *Vegfa* in day-2 wounds between *Langerin*^{DTR} mice pre-treated with saline and DT. ns, not significant. Data are depicted as mean±SEM.

Fig. S7

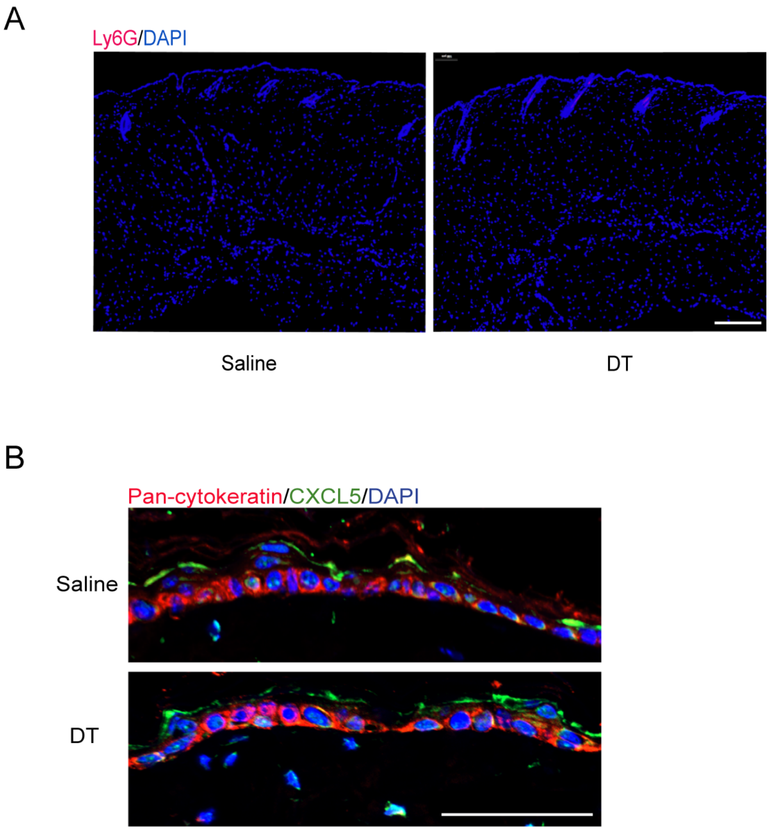

Figure S7. DT-treatment to *Langerin*^{DTR} mice did not cause neutrophil infiltration or induce CXCL5 upregulation in normal backskin. A. Immunostaining of Ly6G showed that there were almost no neutrophils in normal skin in both conditions. Scale bar: 200 µm. **B.** Co-immunostaining of pan-cytokeratin and CXCL5 showed very little CXCL5 expression in both LC-depleted (DT) and LC-present (saline) skin. Scale bar: 50 µm.

Fig. S8

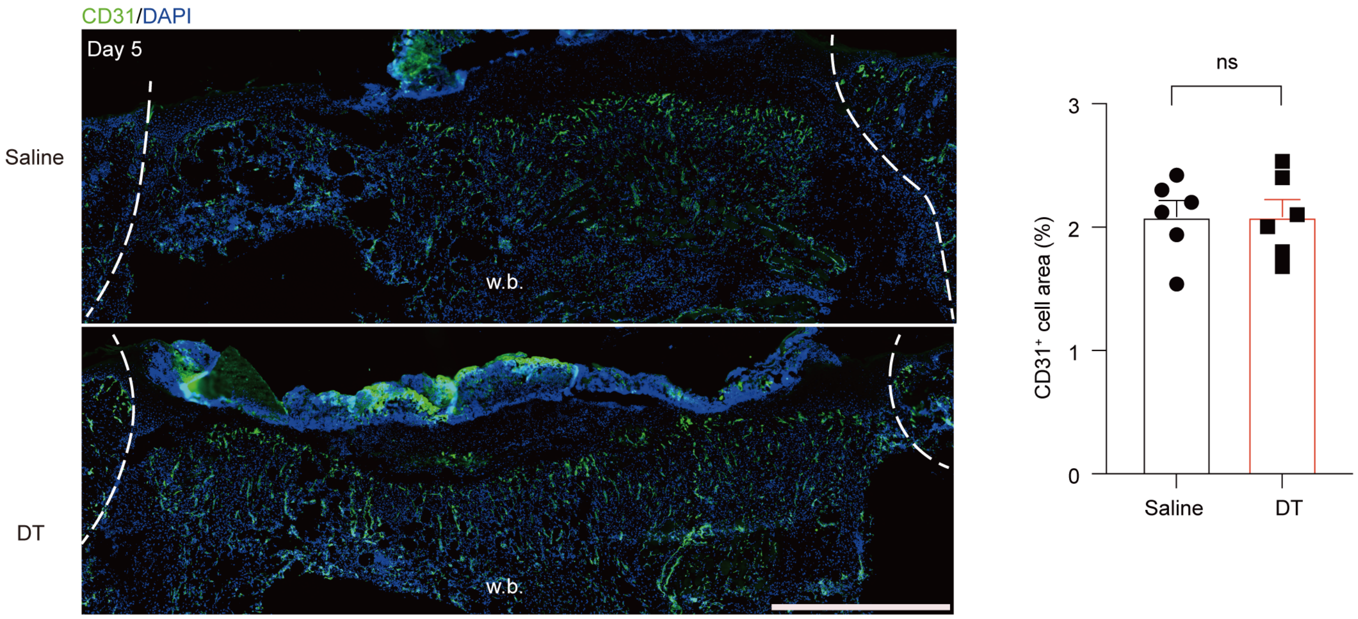

Figure S8. There was no significant difference in the densities of vasculature, as demonstrated by CD31 staining, between DT-treated and saline-treated *Langerin*^{DTR} mice on Day 5 post wounding. Scale bar: 500 µm.

Fig. S9

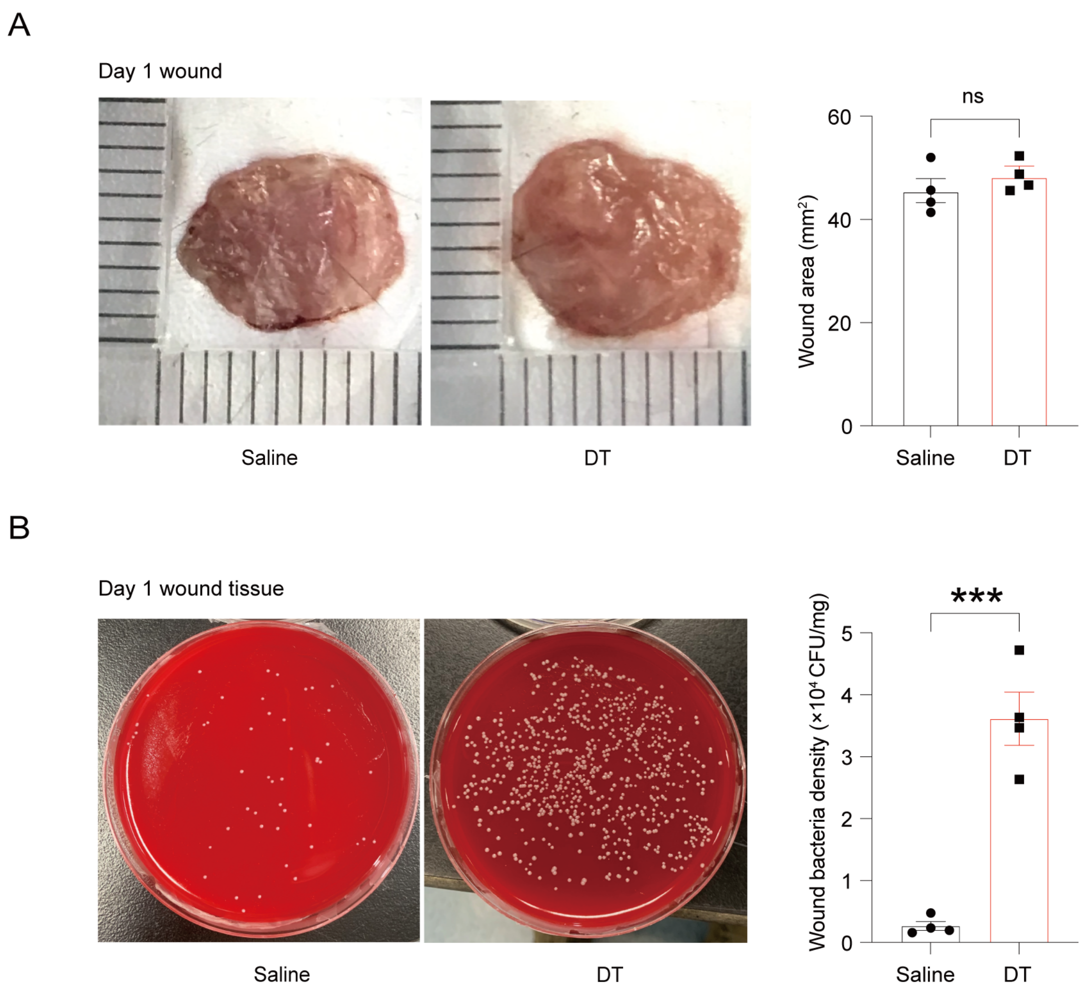

Figure S9. There was no significant difference of day 1 wound area between DT-treated and saline-treated *Langerin*^{DTR} mice (A), but significantly increased number of bacteria in the wounds of LC-deficient mice (DT) than LC-intact mice (saline) on day 1 post wounding. CFU: clone forming unit. ***: $P<0.001$, ns: not significant. Data are depicted as mean±SEM.

Fig. S10

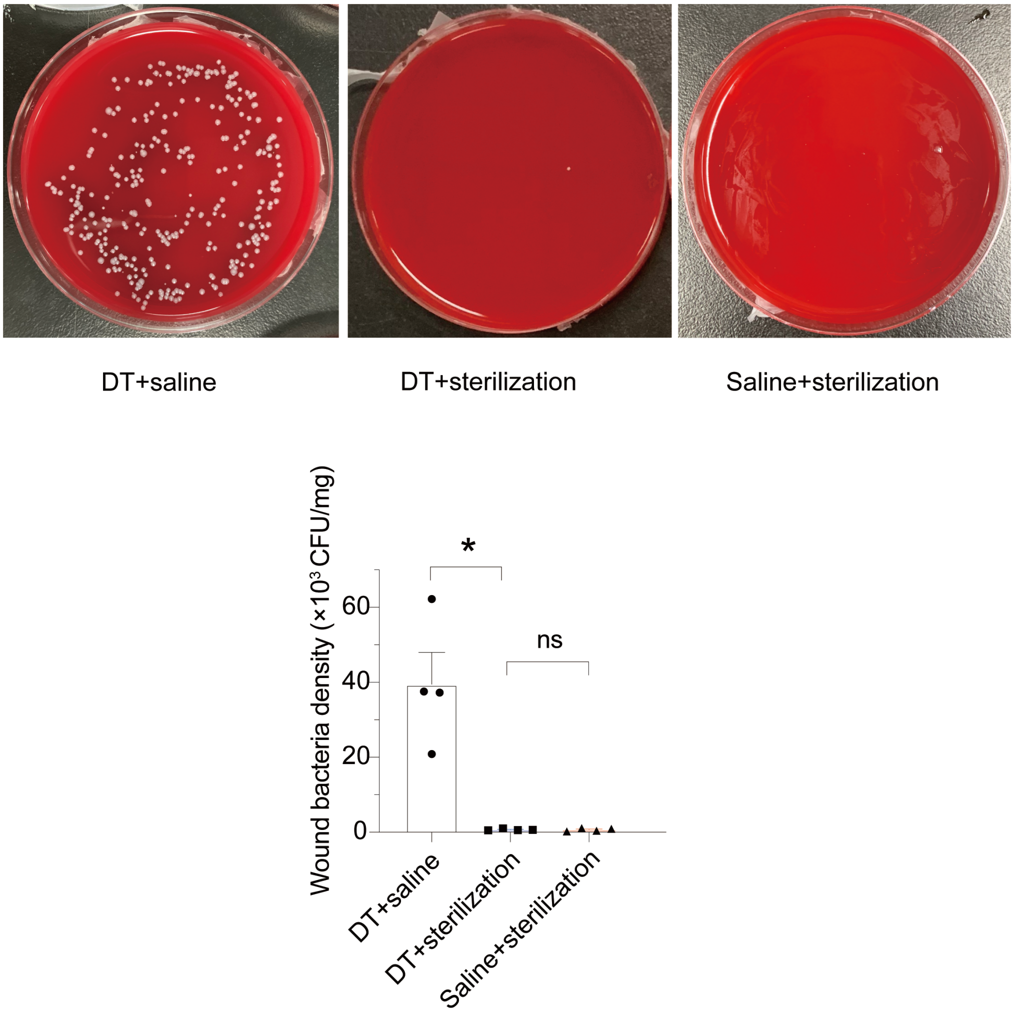

Figure S10. The bacterial load in the wounds of DT-injected mice significantly decreased by applied benzalkonium chloride topically. CFU: clone forming unit. *: $P<0.05$, ns: not significant. Data are depicted as mean±SEM.
